# Taxonomic notes relating to *Glenea diversenotata* Schwarzer and *G. quadriguttata* Pic (Coleoptera, Cerambycidae, Lamiinae)

**DOI:** 10.3897/zookeys.586.7423

**Published:** 2016-05-04

**Authors:** Mei-Ying Lin, Steven W. Lingafelter

**Affiliations:** 1 Key Laboratory of Zoological Systematics and Evolution, Institute of Zoology, Chinese Academy of Sciences, Beichen West Road, Chaoyang Dist., Beijing, 100101, China; 2 Systematic Entomology Lab, ARS, USDA, National Museum of Natural History, MRC-168, P.O. Box 37012, Washington, DC 20013-7012, U. S. A.

**Keywords:** Saperdini, new synonym, lectotype, Taiwan, Oriental region

## Abstract

*Glenea diversenotata* Schwarzer, 1925 is reinstated from a subspecies of *Glenea tonkinea* Aurivillius, 1925 to species level and *Glenea neohumerosa* Lin & Yang, 2011 is a new junior synonym. Some biological information on *Glenea diversenotata* is recorded for the first time, including pictures of the larva and pupa. *Glenea quadriguttata* Pic, 1926 is reinstated from a subspecies of *Glenea lacteomaculata* Schwarzer, 1925 to species level. Lectotypes for *Glenea lacteomaculata* and *Glenea quadriguttata* are designated. A modified key to the related species is presented.

## Introduction

*Glenea diversenotata* Schwarzer, 1925 and *Glenea quadriguttata* Pic, 1926 were described from Taiwan and N. Vietnam, respectively. Later, the former was downgraded as a subspecies of *Glenea tonkinea* Aurivillius, 1925 and the latter as a subspecies of *Glenea lacteomaculata* Schwarzer, 1925 by Breuning (1956). Comparison to the type materials, however, reveals that each of them should be reinstated to species level from subspecies level. We therefore present the new taxonomic decisions, justifications, and full synonymies below.

## Materials

Types and other material studied are deposited in the following institutions:

IZAS
Institute of Zoology, Chinese Academy of Sciences, Beijing, China 


MNHN
Muséum national d’Histoire naturelle, Paris, France 


SMF
 Forschungsinstitut und Naturmuseum Senckenberg, Frankfurt-am-Main, Germany 


SDEI
Senckenberg Deutsches Entomologisches Institut, Müncheberg, Germany 


## Results

### Glenea diversenotata

Taxon classificationAnimaliaColeopteraCerambycidae

Schwarzer, 1925

Glenea diversenotata Schwarzer, 1925: 152. Type locality: China, Taiwan, Kosempo. Type depository: SDEI.
Glenea (*s. str.*) diversenotata; Gressitt 1951: 575.
Glenea (Glenea) tonkinea sbsp. diversenotata; Breuning 1956b: 743; Breuning 1966: 689.
Glenea (Glenea) tonkinea diversenotata ; Yu and Nara 1988: 45, 92, pl. 20, fig. 23; Yu, Nara and Chu 2002: 68, 119, pl. 25, fig. 11; Löbl and Smetana 2010: 327.
Glenea (Glenea) tonkinea subsp. diversenotata; Nakamura, Makihara, Saito 1992: 104; Nakamura et al. 2014: 175.
Glenea diversenotata ; Hua 2002: 210; Behne and Gaedike 2013: 177.
Glenea tonkinea diversenotata ; Hua 2002: 210; Hua et al. 2009, 219, 360 (the picture pl. LXXXIV, fig. 967 is a Glenea coomani Pic).
Glenea neohumerosa Lin & Yang, 2011: 62, figs 12–23. **New synonym**.
Glenea neohumerosa ; Lin 2015a: 204, fig. 205-3; 2015b: 290, figs 1859448, 1859455, 1859450.

#### Remarks.

Breuning (1956) treated *Glenea diversenotata* Schwarzer as a subspecies of *Glenea tonkinea* Aurivillius, 1925. The first author examined a photograph of the holotype of *Glenea diversenotata* Schwarzer, 1925 (taken by Nobuo Ohbayashi, Japan) and a photograph of a live specimen matching it from Taiwan (taken by Yu-Long Lin, Taiwan). Our study of these additional materials necessitates a new synonymy of *Glenea neohumerosa* Lin & Yang, 2011. We can find no morphological differences to maintain them as separate species. Likewise, our examination of the types of *Glenea diversenotata* and *Glenea tonkinea* leads us to review the taxonomic position of them since Breuning (1956) that *Glenea diversenotata* is a subspecies of *Glenea tonkinea*. We can find no morphological support for that treatment by Breuning. They are easily separated from each other by the following characters – *Glenea tonkinea*: vertex and occiput of the head with two separate, longitudinal vittae of white pubescence; white elytral maculae more slender and transverse; outer, basal, white, elytral maculae anterolaterally positioned relative to the larger, basal, sutural maculae; outer apical spine of elytra weakly produced; — *Glenea diversenotata*: vertex and occiput with vittae partially fused, not separate; white elytral maculae more rounded; outer basal elytral maculae parallel with the larger, basal, sutural maculae; outer apical spine of elytra pronounced.

#### Distribution.

China (Fujian, Taiwan, Hainan, Guangxi); Vietnam (Tonkin).

#### Additional specimens examined (Lin and Yang 2011).

Holotype, female (Figs 9a, 9b), Formosa, Kosempo,1912.VI.7, leg. H. Sauter (SDEI); 1 male, Taiwan, Pingdong County, Mt. Dahanshan, 2010. VIII.29, leg. Yu-Long Lin & Wenhsin Lin (examined through a live picture, specimen is deposited in private collection of Yu-Long Lin, Taiwan).

#### Biological notes.

**Figures 1–5.**
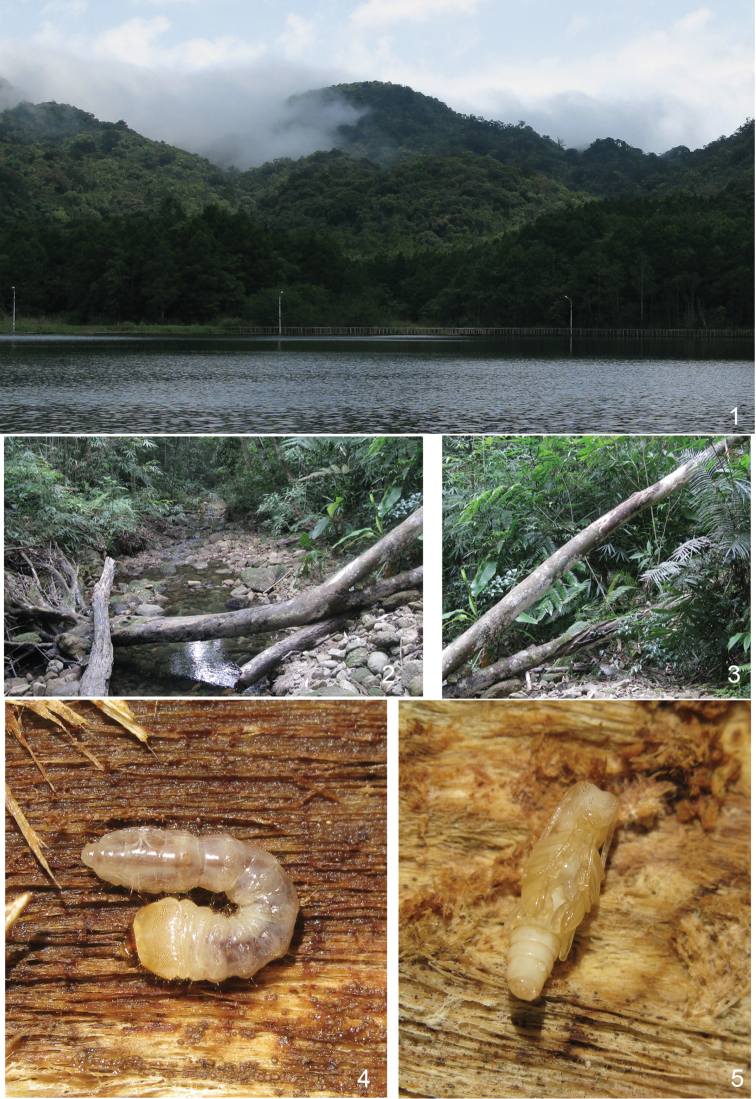
*Glenea diversenotata* Schwarzer, 1925 from Hainan, Diaoluoshan, taken in 2010.IV.23, by Zi-Wei Yin. **1** The broad-leaved forest located in Hainan, Diaoluoshan **2–3** A large decomposing log with *Glenea diversenotata* Schwarzer, 1925 inside **4** Larva **5** Pupa.

**Figures 6–8.**
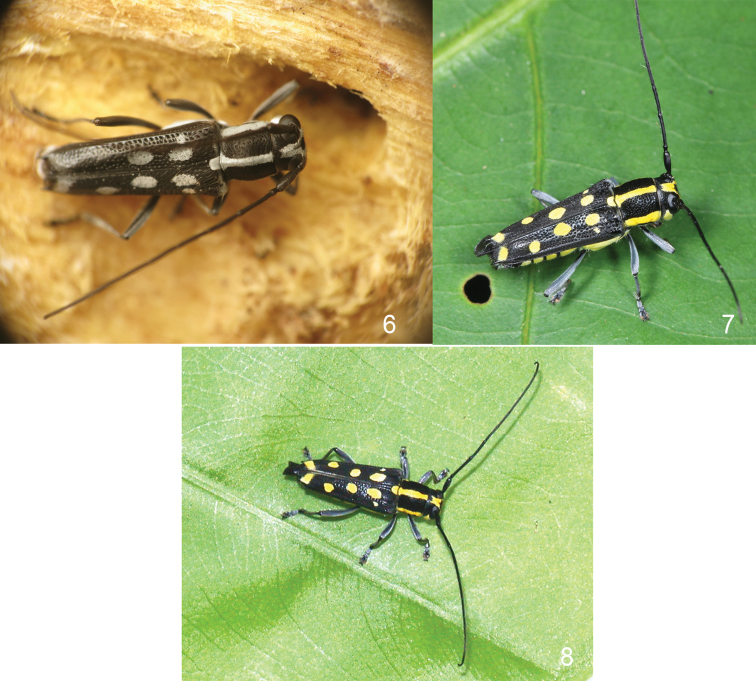
*Glenea diversenotata* Schwarzer, 1925, adult, live pictures. **6** A fresh emerged adult from the log in fig. 2, from Hainan, Diaoluoshan, showing the white pubescence, taken in 2010.IV.23, by Zi-Wei Yin **7** An active adult from Hainan, Jianfengling, showing the yellow pubescence, taken in 2011.V.23, by Wen-Xuan Bi **8** An active adult from Taiwan, Dahanshan, showing the yellow pubescence, taken in 2010.VIII.29, by Yu-Long Lin.

A larva (Fig. 4), two pupae (Fig. 5) and two adults (Fig. 6) were extracted from a large, decomposing log (Figs 2–3) found from a creek valley in a broad-leaved evergreen forest (Fig. 1) in 23 April, 2010 (according to personal communication with Zi-Wei Yin, in December, 2013). The stream is located in Hainan, Lingshui County, Diaoluoshan, ca. 935 m, 18°43'36"N, 100°52'14"E. The fresh emerged adults (Fig. 6) have the pubescence white which becomes yellow afterwards (Figs 7–8), and becoming white again after pinned and dried.

**Figures 9–12.**
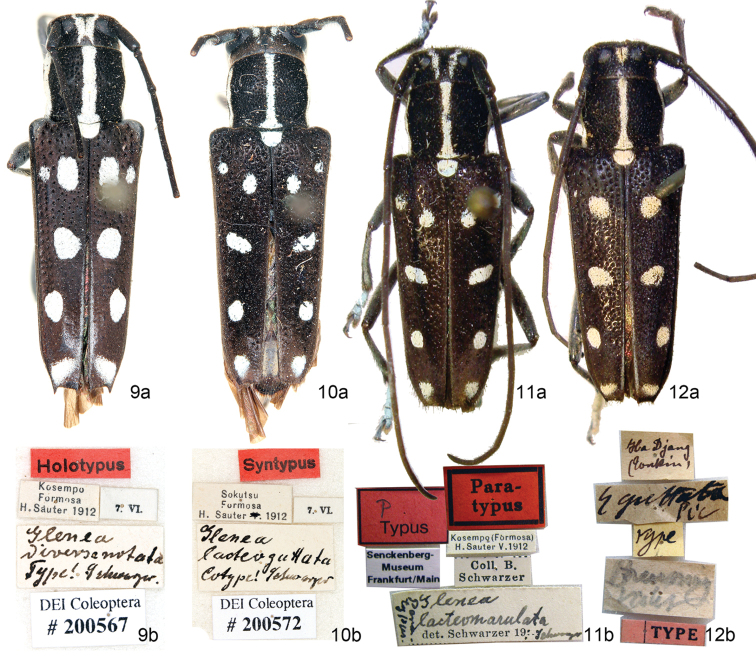
*Glenea* spp., **a** habitus, dorsal view **b** labels **9** *Glenea diversenotata* Schwarzer, 1925, holotype, female, in SDEI (photographed by Nobuo Ohbayashi) **10–11** *Glenea lacteomaculata* Schwarzer, 1925 **10** Lectotype, female, in SDEI (photographed by Nobuo Ohbayashi) **11** paralectotype, female, in SMF (photographed by Mei-Ying Lin) **12** *Glenea quadriguttata* Pic, 1926, lectotype, female, in MNHN (photographed by Mei-Ying Lin). All pictures not to scale.

### Glenea tonkinea

Taxon classificationAnimaliaColeopteraCerambycidae

Aurivillius, 1925

Glenea tonkinea Aurivillius, 1925: 521, fig. 160. Type locality: Vietnam, Tonkin, Hoa Binh. Type depository: MNHN.
Glenea (*s. str.*) tonkinea; Gressitt 1951: 580.
Glenea (Glenea) tonkinea m. basirufofemorata Breuning, 1956a: 698; Breuning 1956b: 743; Breuning 1966: 689.
Glenea (Glenea) tonkinea ; Breuning 1966: 689.
Glenea (Glenea) tonkinea m. apicetruncata Breuning, 1956b: 743; Breuning 1966: 689.
Glenea (Glenea) tonkinea ; Nakamura, Makihara, Saito, 1992: 104; Nakamura et al. 2014: 175.
Glenea tonkinea tonkinea ; Hua, Nara and Yu 1993: 165, 297, pl. XXII, fig. 370b; Hua 2002: 210; Hua et al. 2009, 219, 360, pl. LXXXIV, fig. 967.
Glenea (Glenea) tonkinea tonkinea ; Löbl and Smetana 2010: 327.
Glenea tonkinea ; Lin and Yang 2011: 67, figs 30–33.

#### Remarks.

The record from Taiwan (Nakamura, Makihara, Saito, 1992; Hua 2002; Löbl and Smetana 2010; Nakamura et al. 2014) is doubtful. This taxon was not mentioned in the four volumes of Taiwanese fauna book (Yu and Nara 1988; Yu, Nara and Chu 2002; Chou 2004, 2008).

#### Distribution.

China (Guangdong, Hainan, Guangxi, Taiwan?); Vietnam (Tonkin), Myanmar.

### Glenea lacteomaculata

Taxon classificationAnimaliaColeopteraCerambycidae

Schwarzer, 1925 lectotype designation

Glenea lacteomaculata Schwarzer, 1925: 151. Type locality: China, Taiwan, Kosempo, Sokutsu. Type depository: SDEI.
Glenea (*s. str.*) lacteomaculata; Gressitt 1951: 575.
Glenea (Glenea) lacteomaculata ; Breuning 1956b: 743; Breuning 1966: 689; Yu and Nara 1988: 45, 92, pl. 20, fig. 18; Yu, Nara and Chu 2002: 68, 119, pl. 25, fig. 10.
Glenea (Glenea) lacteomaculata ; Nakamura, Makihara, Saito, 1992: 105; Nakamura et al. 2014: 174.
Glenea lacteomaculata ; Hua 2002: 210; Hua et al. 2009, 216, 358, pl. LXXXII, fig. 939.
Glenea (Glenea) lacteomaculata lacteomaculata ; Löbl and Smetana 2010: 325; Danilevsky 2013: 201.
Glenea lacteomaculata ; Lin and Yang 2011: 65, fig. 24; Behne and Gaedike 2013: 183.

#### Remarks.

Lin and Yang (2011) were unable to define *Glenea lacteomaculata* Schwarzer, 1925. Subsequently, the first author examined a photograph of a syntype of *Glenea lacteomaculata* (taken by Nobuo Ohbayashi, Japan). Comparing that photograph with another of *Glenea quadriguttata* Pic, 1926, it is possible to conclude that the latter must be elevated to species. Hua (2002) indexed Taiwan and Guangxi and this was followed by Löbl and Smetana (2010). However, the specimens from Guangxi should be *Glenea quadriguttata* Pic, 1926 (Lin and Yang 2011) and only Taiwan was the known locality of *Glenea lacteomaculata* Schwarzer, 1925.

#### Distribution.

China: Taiwan.

#### Lectotype designation.

According to the original description (Schwarzer 1925), there were multiple type specimens. The syntypes were deposited in SDEI and SMF. In order to fix the species concept and ensure universal and consistent interpretation of this species, we designate the female specimen in SDEI as the lectotype (Fig. 10a, b), the female in SMF (Fig. 11a, b) and another one with same collecting data to the lectotype (Behne and Gaedike 2013) as the paralectotypes of *Glenea lacteomaculata* Schwarzer, 1925. Though the female in SMF is in better condition than the female with antennae mostly lost in SDEI, we pick up the SDEI one as lectotype according to ICZN Recommendation 74D. The majority of Schwarzer’s types collected by Hans Sauter is contained in SDEI (listed by Behne and Gaedike 2013; Stephan Blank and Junsuke Yamasako, personal communication in November, 2015). The lectotype has the following labels: “Sokutsu/ Formosa/ H. Sauter 1912” printed, “7.VI.” printed, “Glenea lacteomaculata / Cotype! Schwarzer” handwritten, “Syntypus” printed on red label, “DEI Coleoptera # 200572” printed on white label. And a lectotype label will be added by the managers in SDEI after this paper.

### Glenea quadriguttata

Taxon classificationAnimaliaColeopteraCerambycidae

Pic, 1926 revised status, lectotype designation

Glenea 4-guttata Pic, 1926: 22. Type locality: Vietnam, Tonkin, Djang. Type depository: MNHN.
Glenea (Glenea) lacteomaculata sbsp. quadriguttata; Breuning 1956b: 743, 744; Breuning 1966: 689.
Glenea lacteomaculata quadriguttata ; Lin and Yang 2011: 65, figs 25–26.
Glenea (Glenea) lacteomaculata quadriguttata ; Danilevsky 2013: 201.

#### Remarks.

Breuning (1956) had previously treated *Glenea quadriguttata* as a subspecies of *Glenea lacteomaculata*. We have found the following morphological differences between them — *Glenea lacteomaculata*: vertex and occiput of the head with two separate, longitudinal vittae of white pubescence; punctures at the base of the elytra denser and more irregularly spaced than in *Glenea quadriguttata*; central-most, white elytral maculae more slender, transverse and oblique; elytral apex without acute or toothed angles; — *Glenea quadriguttata*: head with vittae fused, not separate; central-most, white elytral maculae almost rounded; elytral apex with small sutural and outer apical teeth. With these differences, *Glenea quadriguttata* is reinstated as an independent species.

#### Distribution.

China (Guangxi, Yunnan); Vietnam (Tonkin).

#### Lectotype designation.

The original description does not allow to know the number of specimens used by Pic (1926). In order to fix the species concept and ensure universal and consistent interpretation of this species, we designate the female specimen with Pic’s handwriting labels as the lectotype (Fig. 12a, b), and the first author did not find another syntype to be paralectotype when she worked in MNHN in 2007–2008. The lectotype has the following labels: “Ha Djang/ (Tonkin)” handwritten, “Glenea quadriguttata Pic” handwritten, “type” handwritten on yellow label, “Breuning valiv” handwritten, “TYPE” printed on pink red label. And a lectotype label will be added by the managers in MNHN after this paper.

### Key to *Glenea coomani* group

(modified from Lin and Yang 2011)

**Table:** 

1	Elytral apex having only a short tooth at the outer angle (subequal to that at the sutural angle, fig. 34 in Lin and Yang 2011)	**2**
–	Elytral apex having a long and sharp spine at the outer angle (much longer than that at the sutural angle, fig. 35 in Lin and Yang 2011)	**5**
2	Elytron having only one big oval macula at basal quarter; the second macula is the smallest (Figs 1–4 in Lin and Yang 2011)	***Glenea coomani***
–	Elytron having two small spots at basal quarter, the first spot is the smallest	**3**
3	Vertex with one pubescent spot between upper eye lobes; the middle spot on elytron almost rounded (Fig. 12)	***Glenea quadriguttata***
–	Vertex with two pubescent vittae between upper eye lobes; the middle spot on elytron transverse and oblique	**4**
4	Suture without pubescence stripe; the middle vitta shorter and still far from suture; the apical pubescent vitta small and with distance from suture (Figs 10–11)	***Glenea lacteomaculata***
–	Suture with pubescence stripe; the middle vitta longer and almost touching sutural stripe; the apical pubescent vitta larger and fused with sutural stripe (Figs 30–33 in Lin and Yang 2011)	***Glenea tonkinea***
5	Elytron having 5 white or yellow maculae (figs 12–15 in Lin and Yang 2011; Figs 6–9)	***Glenea diversenotata***
–	Elytron having 6 white or yellowbrown maculae	**6**
6	Legs testaceous; elytral apical spot smaller, not touching suture; vertex with two yellowish-brown spots between upper eye lobes (fig. 27 in Lin and Yang 2011)	***Glenea laodice***
–	Legs black; elytral apical spot larger and touching suture; vertex with one yellowish-brown spot between upper eye lobes (figs 28–29 in Lin and Yang 2011)	***Glenea subalcyone***

## Supplementary Material

XML Treatment for Glenea diversenotata

XML Treatment for Glenea tonkinea

XML Treatment for Glenea lacteomaculata

XML Treatment for Glenea quadriguttata
